# LncRNA AC093818.1 accelerates gastric cancer metastasis by epigenetically promoting PDK1 expression

**DOI:** 10.1038/s41419-020-2245-2

**Published:** 2020-01-27

**Authors:** Ming-chen Ba, Zheng Ba, Hui Long, Shu-zhong Cui, Yuan-feng Gong, Zhao-fei Yan, Kun-peng Lin, Yin-bing Wu, Yi-nuo Tu

**Affiliations:** 1 Intracelom Hyperthermic Perfusion Therapy Center, Affiliated Cancer Hospital & Institute of Guangzhou Medical University, Guangzhou, 510095 P.R. China; 2 Intensive Care Unit, Zhujiang Hospital, Southern Medical University, Guangzhou, 510282 China; 3 Department of Pharmacy, Guangzhou Dermatology Institute, Guangzhou, 510095 P.R. China

**Keywords:** Cancer of unknown primary, Diagnostic markers

## Abstract

Gastric cancer (GC) is a highly prevalent type of metastatic tumor. The mechanisms underlying GC metastasis are poorly understood. Some long noncoding RNAs (lncRNAs) reportedly play key roles in regulating metastasis of GC. However, the biological roles of five natural antisense lncRNAs (AC093818.1, CTD-2541M15.1, BC047644, RP11-597M12.1, and RP11-40A13.1) in GC metastasis remain unclear. In this study, the expression of these lncRNAs was measured by quantitative reverse transcription-polymerase chain reaction. Migration and invasion were evaluated by wound-healing and the Transwell assay, respectively. Stable cells were injected into the tail veins of nude mice. Sections of collected lung and liver tissues were stained using hematoxylin and eosin. Protein expression was analyzed by western blot. RNA immunoprecipitation (RIP) assay was used to verify whether the STAT3 and SP1 transcription factors bound to AC093818.1 in GC cells. Expression levels of the five lncRNAs, especially AC093818.1, were significantly upregulated in metastatic GC tissues relative to those in nonmetastatic GC tissues. AC093818.1 expression was correlated with invasion, lymphatic metastasis, distal metastasis, and tumor-node-metastasis stage. AC093818.1 expression was highly sensitive and specific in the diagnosis of metastatic or nonmetastatic GC. AC093818.1 overexpression promoted GC migration and invasion in vitro and in vivo. AC093818.1 overexpression increased PDK1, p-AKT1, and p-mTOR expression levels. AC093818.1 silencing decreased these expressions. AC093818.1 bound to transcription factors STAT3 and SP1, and SP1 or STAT3 silencing could alleviated the effect of AC093818.1 overexpression. The data demonstrate that lncRNA AC093818.1 accelerates gastric cancer metastasis by epigenetically promoting PDK1 expression. LncRNA AC093818.1 may be a potential therapeutic target for metastatic GC.

## Introduction

Gastric cancer (GC) is one of the most common cancers worldwide^1^. Compared to most countries, GC-associated morbidity and mortality are generally higher in East Asia, particularly in Korea, Mongolia, Japan, and China^2,3^. In recent years, the incidence of GC has decreased with the development of early detection methods^4^. Treatment at the early stages of GC by complete surgical resection has been associated with good prognosis^5^. However, metastatic GC has a poor 5-year survival rate of approximately 30%^6^. GC is a highly prevalent metastatic tumor, and the mechanisms of metastasis are poorly understood.

Long noncoding RNAs (lncRNAs) are a class of newly discovered noncoding RNA molecules that are more than 200 nucleotides in length^7^. LncRNAs are involved in a wide range of biological processes including cell proliferation, metastasis, differentiation, inflammation, and angiogenesis^8–10^. An increasing number of recent studies have revealed that lncRNAs participate in the progression of GC by regulating metastasis and angiogenesis^11^. The ncRNAs Z38^12^, LINC01606^13^, and SNHG16 regulate cell metastasis in GC. LINC01410^14^, PVT1^15^, and MALAT1^16^ contribute to the regulation of vasculogenic mimicry and angiogenesis, which can in turn aggravate GC progression. Song et al. identified 2710 differentially regulated lncRNAs in lymph node-metastasized samples (1381 upregulated and 1329 downregulated) relative to primary GC samples^17^. However, the effects of these lncRNAs on GC metastasis remain unclear.

In the present study, we investigated the expression of five upregulated natural antisense lncRNAs (AC093818.1, CTD-2541M15.1, BC047644, RP11-597M12.1, and RP11-40A13.1), that were previously identified^17^, and evaluated their role in GC metastasis. We collected metastatic and nonmetastatic GC tissues and analyzed their lncRNA expression patterns. AC093818.1 was selected for further study. The antisense transcript of AC093818.1 is phosphoinositide-dependent kinase-1 (PDK1)^17^. We assessed the relationship between AC093818.1 expression and its diagnostic power and clinical significance in GC. Additionally, we investigated the role and underlying mechanism of AC093818.1 in GC metastasis. Our findings highlight the important role of AC093818.1 in GC metastasis and its potential as a therapeutic target for GC.

## Materials and methods

### Patients and tissue collection

A total of 85 tissue samples were obtained from patients with metastatic GC, and 20 tissue samples were obtained from patients with nonmetastatic GC. The patients were recruited from January 2015 to December 2017 from the Affiliated Cancer Hospital & Institute of Guangzhou Medical University before receiving chemotherapy or radiation therapy. The inclusion criteria for enrollment in the study were: (1) GC patients aged ≥ 18 years; (2) diagnosis of GC via gastric endoscopy, computed tomography, and/or magnetic resonance imaging; and (3) metastasis confirmed via B-ultrasound, computed tomography, magnetic resonance imaging, and/or serum tumor biomarker examination. Patients satisfying any of the following criteria were excluded: (1) benign gastric tumors; (2) gastric nonepithelial malignant tumors; (3) GC patients unsuitable for operations because of extensive abdominal adhesions, or (4) GC patients unsuitable for surgical resection because of tumor extensive organ infiltration. Diagnosis and clinicopathological characteristics including age, gender, tumor size, differentiation, invasion, lymphatic metastasis, distal metastasis, and tumor-node-metastasis (TNM) stage were confirmed by two pathologists. All tissues were stored at −80 °C until further processing. All participants signed informed consent forms. All experiments were approved by the Ethics Committee of the Affiliated Cancer Hospital & Institute of Guangzhou Medical University.

### RNA extraction and quantitative reverse transcription-polymerase chain reaction (qRT-PCR)

Total RNA was extracted from gastric tissues and cells using TRIzol reagent (Invitrogen, Carlsbad, CA, USA) and reverse-transcribed into cDNA using PrimeScript RT Reagent kit with cDNA Eraser (TaKaRa Bio, Dalian, China). LncRNA expression levels were measured by qRT-PCR using SYBR Premix Ex Taq (TaKaRa Bio) on a 7500 real-time PCR system (Applied Biosystems, Foster City, CA, USA). Primer sequences used for qRT-PCR are shown in Supplementary Table 1. Glyceraldehyde 3-phosphate dehydrogenase (GAPDH) was used as an internal control to normalize lncRNA expression. Relative lncRNA expression was expressed as fold-change following the 2^−ΔΔCt^ method. qRT-PCR assays were replicated three times.

### Cell culture

BGC823, MGC803, MKN28, MKN45, and SGC7901 human GC cell lines were obtained from the Type Culture Collection of the Chinese Academy of Sciences (Shanghai, China). All cells were recently authenticated by Short Tandem Repeat profiling and tested for mycoplasma contamination. Cells were cultured in Dulbecco’s modified Eagle’s medium (HyClone, Logan, UT, USA) or RPMI-1640 medium (HyClone) containing 10% fetal bovine serum (FBS; Gibco, Logan, UT, USA) and 100 U of penicillin and streptomycin at 37 °C in a humidified atmosphere with 5% CO_2_.

### Synthesis of small interfering RNAs (siRNAs)

The siRNAs were synthesized by GenePharma Co., Ltd. (Shanghai, China). These included three siRNAs targeting AC093818.1 (siRNA-1 sense sequence: ACCAAGAGGCTGAAATGAAAG, siRNA-2 sense sequence: GTAGGCTACCTCTTTACTAAC, siRNA-3 sense sequence: TCTGATTATCAGATTAGATTA, siRNA-4 sense sequence: CAAAGAAGCAAAUACACUAAA, siRNA-5 sense sequence: GAUUAUCAGAUUAGAUUAAGA, siRNA-6 sense sequence: CAGAUUAGAUUAAGAAUAAUU), one siRNA targeting SP1 (si-SP1, sense sequence GGAUGGUUCUGGUCAAAUACA), one siRNA targeting signal transducer and activator of transcription 3 (si-STAT3, sense sequence: GCCUGUUUCUGUAAGCAAAUG), and a negative control siRNA (si-NC, sense sequence: UUCUCCGAACGUGUCACGUTT).

### Lentivirus packaging and generation of stable cells

The AC093818.1 sequence was amplificated using the primers shown in Supplementary Table 1 and inserted into the PLVX-IRES-NEO-EGFP plasmid. The short hairpin RNA (shRNA) of AC093818.1 (sense sequence: GATCCTCTGATTATCAGATTAGATTATTCAAGAGATAATCTAATCTGATAATCAGATTTTTTG) (sh-AC093818.1) was designed according to the targeting sequence of siRNA-3 on AC093818.1 and inserted into the pLVX-IRES-ZsGreen1 plasmid. Lentivirus packaging was performed according to a previously described method^18^. Lentivirus overexpressing AC093818.1 was denoted as ov-AC093818.1 and the negative control lentivirus was denoted as ov-NC. Lentivirus expressing sh-AC093818.1 was denoted as sh-AC093818.1 and the negative control lentivirus was denoted as sh-NC. MKN28 cells were infected with ov-AC093818.1 and ov-NC, and MGC803 cells were infected with sh-AC093818.1 and sh-NC. Generation of stable cells was performed according to a previously described method with minor revision^18^. Briefly, infected cells were passaged twice per week in complete medium containing 1 μg/mL puromycin. After 1 week, cells were screened using limiting dilution. After culture in complete medium containing puromycin for 2 weeks, cells were further screened by two limiting dilutions to obtain stable cell lines. The stable cell lines were then bulk cultured for subsequent assays.

### Transwell assay

Cell migration and invasion were evaluated by conducting the Transwell assay, according to a previously described method^19,20^. Transwell inserts (pore size, 8 μm; BD Biosciences, San Jose, CA, USA) were precoated with or without Matrigel (BD Biosciences) for use in the migration and invasion assays. Infected MGC803 and MKN28 cells were harvested and 1 × 10^5^ cells were suspended in 100 μL of serum-free medium and placed in a Transwell insert. The lower chamber was filled with 600 μL of medium containing 10% FBS. After the cells were incubated for 24 h at 37 °C, cells on the underside of the membrane were fixed with 4% paraformaldehyde for 15 min and subsequently stained with 0.1% crystal violet in 20% ethanol. Cell counts were performed for five randomly selected fields at ×200 magnification using phase contrast microscopy (Olympus, Hamburg, Germany). Transwell assays were replicated three times.

### Wound-healing assay

Cell migration was determined by a wound-healing assay, according to a previously described method^21^. Infected MGC803 and MKN28 cells were seeded into six-well plates at 5 × 10^5^ cells/well. Upon reaching 90% confluence, the plate containing the cells was scratched with a 200-μL pipette tip. The remaining cells were washed with medium and incubated in an atmosphere containing 5% CO_2_ at 37 °C for 24 h. The migration distance of the cells was captured using a FSX100 Biological Image system (Olympus). Images were analyzed using Image version 6.0 (Media Cybernetics, Rockville, MD, USA). Wound-healing assays were replicated three times.

### Western blot

Western blot was performed according to a previously described method^18^. GAPDH was used as an internal loading control. Densitometric analysis was performed on western blot images using Image Pro-Plus 6.0 software (Media Cybernetics). The catalog numbers and dilution of the antibodies used (Abcam, Cambridge, MA, USA) are as follows: matrix metalloproteinase (MMP)-2, ab37150, 1:500; MMP-9, ab38898, 1:1000; E-cadherin, ab15148, 1:500; vimentin, ab92547, 1:2500; PDK1, 1:800, ab110025; p-AKT1 (phospho S473), 1:2500, ab194201; p-mTOR (phospho S2448), 1:1000, ab109268; AKT1, 1:1000, ab227100; mTOR, ab2732, 1:2000; and GAPDH, ab9485, 1:2500. Western blot assays were replicated three times.

### In vivo metastasis assay

Infected MGC803 and MKN28 cell suspensions (1 × 10^6^ cells/200 μL) were injected into the tail veins of 4- to 6-week-old nude mice (*n* = 10, total of 40 mice). The mice were sacrificed at 8 weeks after inoculation. The lungs and livers were collected, fixed in 4% formaldehyde, embedded in paraffin, and sectioned (5 μm). Serial sections were stained with hematoxylin−eosin and imaged using different objective lenses. All animal protocols were approved by the ethics committee of the Affiliated Cancer Hospital & Institute of Guangzhou Medical University and the disposal methods followed the animal ethics standards. A method of randomization was used to determine how animals were allocated to experimental groups and processed, and the investigator was blinded to the group allocation during the experiment.

### RNA immunoprecipitation (RIP) assay

RIP assay was carried out by using the EZMagna RIP kit (Millipore, Bedford, MA, USA). In brief, infected MGC803 and MKN28 cells were lysed and the lysates were incubated with magnetic beads conjugated with anti-SP1 or anti-STAT3 monoclonal antibody, or control IgG (Abcam). The beads were incubated with Proteinase K to elute protein and RNAs from the beads. Finally, RNA was purified and AC093818.1 level was examined by qRT-PCR analysis and PCR products were run on a 3% agarose gel. Fold Enrichment method (2^−ΔΔCt[ChIP/NIS]^) was used to calculate the AC093818.1 level. RIP assays were replicated three times.

### RNA-protein pull-down assay

MAXIscript™ T7 Transcription Kit (AM1312, Invitrogen) was used for the in vitro synthesis of AC093818.1 transcripts or antisense AC093818.1. Thermo Fisher Scientific Pierce RNA 3′ End Desthiobiotinylation Kit was used to attach a single desthiobiotinylated cytidine bisphosphate to the 3′ end of AC093818.1 RNA strand using T4 RNA ligase. Pierce™ Magnetic RNA-Protein Pull-Down Kit to enrich RNA-binding proteins (RBPs) of labeled AC093818.1 using cell lysate of MGC803 and MKN28 cells. The levels of SP1 and STAT3 in enriched RBPs were examined by western blot performed according to a previously described method^18^. The catalog numbers and dilution of the SP1 and STAT3 antibodies (Abcam) are as follows: SP1, 1:4000, ab13370; STAT3, 1:5000, ab119352. RNA-protein pull-down assays were replicated three times.

### PDK1 promotor luciferase assay

PDK1 promotor fragment (−875 to +82)^22^ was amplified from genomic DNA of MGC803 cells and cloned into pGL3-basic vector (Promega, Madison, WI, USA). The product was designated as the pGL3-PDK1 promotor. The full length of SP1 or STAT3 open reading frame was amplified from cDNA of MGC803 cells and cloned into pcDNA3.1 (Invitrogen) to construct SP1 or STAT3 overexpressing plasmid. The product was designated pcDNA-SP1 or pcDNA-STAT3, respectively. MGC803-sh-NC, MGC803-sh-AC093818.1, MKN28-ov-NC, and MKN28-ov-AC093818.1 cells were seeded into 96-well plates and transfected with luciferase reporter plasmid pGL3-PDK1 promotor along with control vector pRL-SV40, pcDNA-SP1, or pcDNA-STAT3. Cell lysates were prepared from cells after 48 h. Firefly luciferase activity was normalized with Renilla luciferase activity (F/R). PDK1 promotor luciferase assay were replicated three times.

### Statistical analyses

Statistical analyses were performed using SPSS 19.0 software (SPSS, Inc., Chicago, IL, USA). Continuous variables are presented as mean ± standard deviation. Differences between two groups were analyzed using an independent *t* test. Differences among more than two groups were evaluated using one-way analysis of variance, followed by least significant difference post-hoc test. A receiver operating characteristic (ROC) curve was used to evaluate the diagnostic performance (sensitivity and specificity) to differentiate between metastatic or nonmetastatic GC. *P* < 0.05 was considered statistically significant.

## Results

### AC093818.1 is strongly upregulated in metastatic GC

AC093818.1, CTD-2541M15.1, BC047644, RP11-597M12.1, and RP11-40A13.1 levels were significantly upregulated in metastatic GC tissues relative to those in nonmetastatic GC tissues (Supplementary Fig. 1). Among the five analyzed lncRNAs, AC093818.1 showed the strongest upregulation (*P* < 0.001) and was therefore used in subsequent experiments.

### AC093818.1 is correlated with clinicopathological features of metastatic GC

**Fig. 1:**
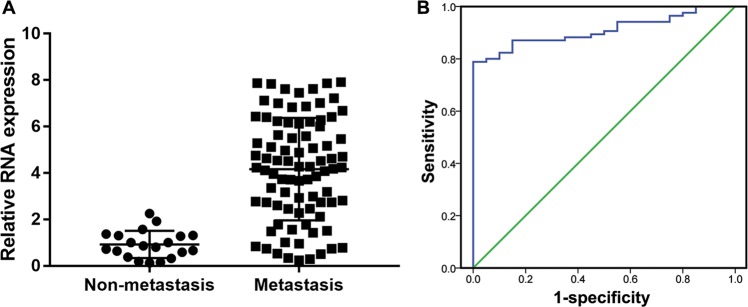
LncRNA AC093818.1 epression in GC tissues and it’s significance in GC metastasis. **a** AC093818.1 expression measured in 20 nonmetastatic GC and 85 metastatic GC samples by qRT-PCR. **b** The performance of the AC093818.1-based model for the diagnosis of metastatic or nonmetastatic GC evaluated by ROC curve analysis.

AC093818.1 expression was significantly higher in metastatic GC tissues than in nonmetastatic GC tissues (Fig. 1a). In particular, AC093818.1 expression showed no correlation with age, gender, tumor size, and differentiation but was significantly correlated with invasion, lymphatic metastasis, distal metastasis, and TNM stage (Table 1). We analyzed the diagnostic performance of AC093818.1 for the diagnosis of metastatic or nonmetastatic GC by conducting ROC curve analysis. At the optimal expression cutoff value of 2.265, the sensitivity of the prediction model based on AC093818.1 was 78.8% and the specificity was 100%, with an area-under-the-curve of 0.908 (95% confidence interval: 0.854–0.963) (Fig. 1b).

**Table 1:** Association between AC093818.1 expression and clinicopathological features of GC patients.

Characteristics	*n* = 85	AC093818.1 expression level	*P*
Age			0.547
≥60	41	4.01 ± 1.915	
<60	44	4.30 ± 2.458	
Gender			0.542
Male	57	4.06 ± 2.258	
Female	28	4.37 ± 2.115	
Tumor size			0.282
≥5	31	4.51 ± 2.046	
<5	54	3.97 ± 2.286	
Differentiation			0.246
Well + moderate	33	3.81 ± 2.293	
Poor	52	4.39 ± 2.139	
Invasion			0.008
T1 + T2	18	2.96 ± 2.008	
T3 + T4	67	4.49 ± 2.155	
Lymphatic metastasis			<0.001
No	30	2.48 ± 1.746	
Yes	55	5.08 ± 1.867	
Distal metastasis			<0.001
M0	71	3.62 ± 1.965	
M1	14	6.94 ± 0.822	
TNM stage			0.001
I + II	28	2.92 ± 1.997	
III + IV	57	4.78 ± 2.052	

### Successful generation of stable AC093818.1-knockdown and overexpressing cells

AC093818.1 expression was the highest in MGC803 and the lowest in MKN28 cells among the five GC cell lines evaluated (BGC823, MGC803, MKN28, MKN45, and SGC7901) (Supplementary Fig. 2A). For AC093818.1 knockdown, MGC803 cells were transfected with three AC093818.1-targeting siRNAs. The qRT-PCR results showed that transfection with the three siRNAs significantly inhibited AC093818.1 expression in MGC803 cells compared to that in si-NC-transfected MGC803 cells (Supplementary Fig. 2B). Among the three AC093818.1-targeting siRNAs, the inhibitory effect of siRNA-3 on AC093818.1 expression was the most obvious. Thus, the sequence of siRNA-3 was chosen to construct the shRNA of AC093818.1.

The expression of AC093818.1 was significantly downregulated in sh-AC093818.1-infected MGC803 cells relative to that in sh-NC-infected MGC803 cells Moreover, AC093818.1 expression was significantly upregulated in ov-AC093818.1-infected MKN28 cells relative to that in ov-NC-infected MKN28 cells (Supplementary Fig. 2C). These stable cell lines were used for subsequent assays.

### AC093818.1 promotes migration and invasion of GC cells

**Fig. 2:**
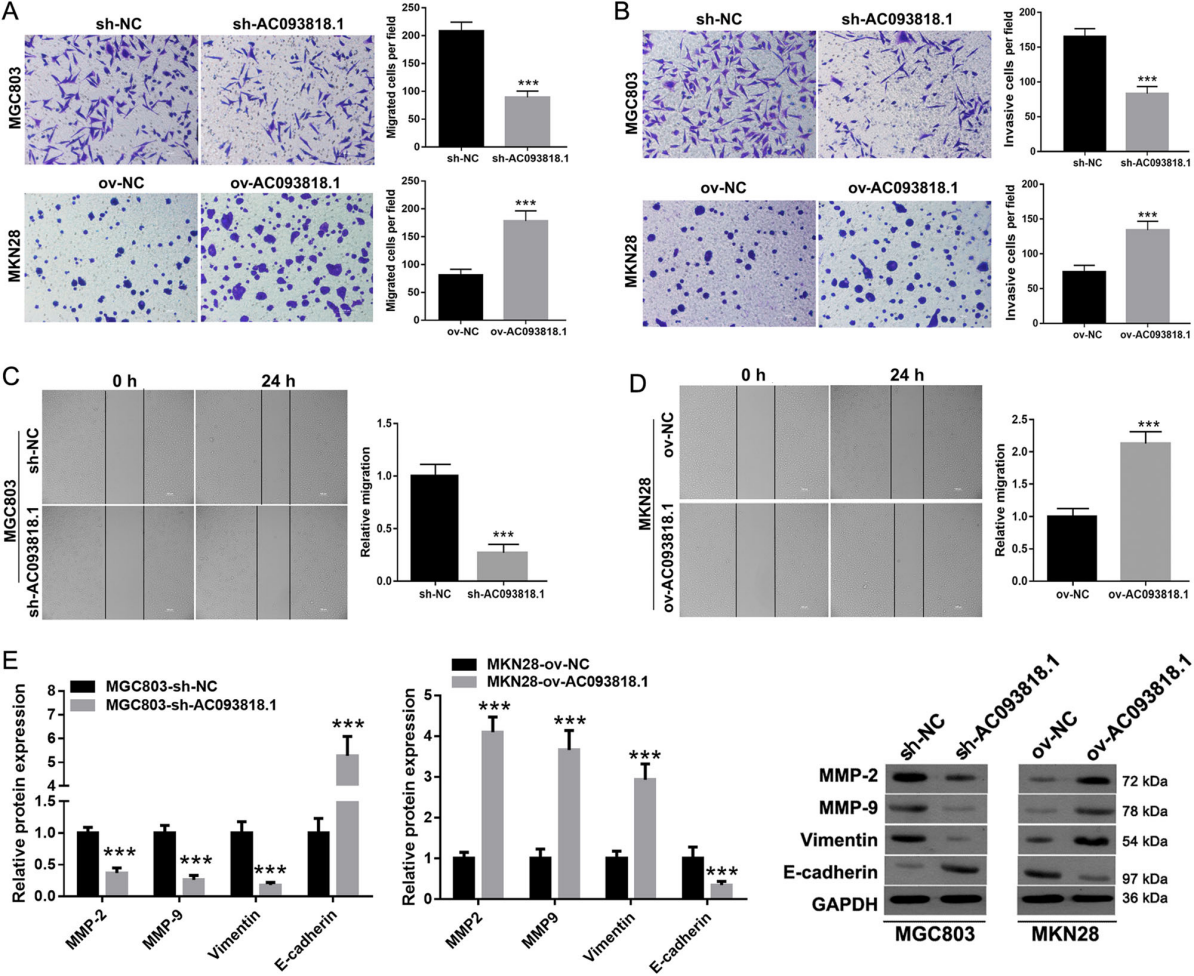
The effect of AC093818.1 knockdown or overexpression on migration and invasion of GC cells in vitro. **a**, **b** Cell migration (**a**) and invasion (**b**) analyzed by the Transwell assay in sh-AC093818.1- and sh-NC-infected MGC803 cells, and ov-AC093818.1- and ov-NC-infected MKN28 cells. The amplification for the left representative images is ×200. **c**, **d** Cell migration analyzed by wound-healing assay in sh-AC093818.1- and sh-NC infected MGC803 (**c**), and ov-AC093818.1 and ov-NC-infected MKN28 cells (**d**). The amplification for the left representative images is ×100. **e** Expression of MMP-2, MMP-9, vimentin, and E-cadherin analyzed by western blot in sh-AC093818.1- and sh-NC-infected MGC803 cells and ov-AC093818.1- and ov-NC-infected MKN28 cells in vitro. ***P < 0.001.

The migration and invasion ability of ov-AC093818.1-infected MKN28 cells was significantly stronger than that of ov-NC-infected MKN28 cells (Fig. 2a–d). Correspondingly, sh-AC093818.1-infected MGC803 cells showed significantly reduced migration and invasion ability relative to those of sh-NC-infected MGC803 cells (Fig. 2a–d). The expression levels of MMP-2, MMP-9, and vimentin were markedly downregulated in sh-AC093818.1-infected MGC803 cells relative to those in sh-NC-infected MGC803 cells, and E-cadherin expression was upregulated in sh-AC093818.1-infected MGC803 cells (Fig. 2e). Additionally, the expression levels of MMP-2, MMP-9, and vimentin were markedly upregulated in ov-AC093818.1-infected MKN28 cells relative to those in ov-NC-infected MKN28 cells, and E-cadherin expression was downregulated in ov-AC093818.1-infected MKN28 cells (Fig. 2e).

To avoid potential off-target effect, we designed other three siRNAs (siRNA-4, -5, -6) to silence AC093818.1 in MGC803 cells. As shown in Supplementary Fig. 2D, the inhibitory effect of siRNA-4 on AC093818.1 expression was the most obvious. Following, we found that siRNA-4 transfection inhibited migration and invasion of MGC803 cells (Supplementary Fig. 3A, B), downregulated expression levels of MMP-2, MMP-9, and vimentin, and upregulated expression levels of E-cadherin (Supplementary Fig. 3C).

### AC0938*1*8.1 induces distant metastasis

**Fig. 3:**
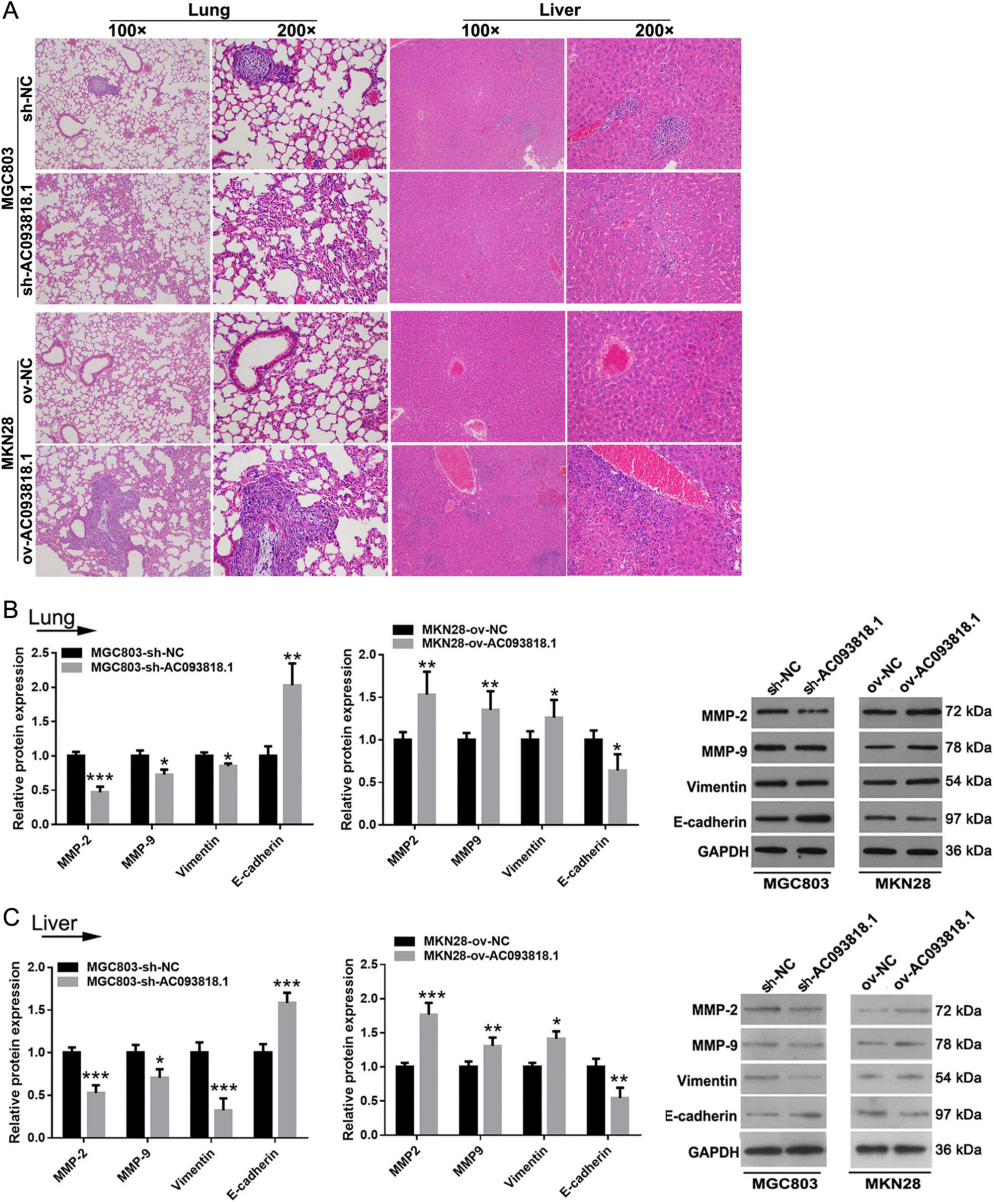
The effect of AC093818.1 knockdown or overexpression on distant metastasis of GC cells in vivo. **a** Hematoxylin and eosin staining of sections prepared from lungs and livers of mice implanted with sh-AC093818.1- and sh-NC-infected MGC803 cells or ov-AC093818.1- and ov-NC-infected MKN28 cells. **b**, **c** Expression of MMP-2, MMP-9, vimentin, and E-cadherin in **b** lung and **c** liver analyzed by western blot in sh-AC093818.1- and sh-NC-infected MGC803 cells, and ov-AC093818.1- and ov-NC-infected MKN28 cells in vivo. *P < 0.05. **P < 0.01. ***P < 0.001.

As shown in Table 2 and Fig. 3a, the incidence of lung and liver metastasis in mice implanted with sh-AC093818.1-infected MGC803 cells was lower than that in mice implanted with sh-NC-infected MGC803 cells. Correspondingly, the incidence of lung and liver metastasis in mice implanted with ov-AC093818.1-infected MKN28 cells was higher than that in mice implanted with ov-NC-infected MKN28 cells (Table 2 and Fig. 3a). The expression levels of MMP-2, MMP-9, and vimentin in lung and liver tissues of mice implanted with ov-AC093818.1- infected MKN28 cells were higher than those of mice implanted with ov-NC-infected MKN28 cells, whereas E-cadherin expression was downregulated (Fig. 3b, c). In addition, the expression levels of MMP-2, MMP-9, and vimentin in lung and liver tissues of mice implanted with sh-AC093818.1-infected MGC803 cells were lower than those of mice implanted with sh-NC-infected MGC803 cells, whereas E-cadherin expression was upregulated (Fig. 3b, c).

**Table 2:** Incidence of lung and liver metastasis of mice implanted with sh-AC093818.1- and sh-NC-infected MGC803 cells and ov-AC093818.1- and ov-NC-infected MKN28 cells.

	Lung and liver metastasis
sh-NC-infected MGC803 cells	9/10
sh-AC093818.1-infected MGC803 cells	3/10
ov-NC-infected MKN28 cells	1/10
ov-AC093818.1- infected MKN28 cells	9/10

### Effect of AC093818.1 on the expression of PDK1, AKT1, and mTOR

**Fig. 4:**
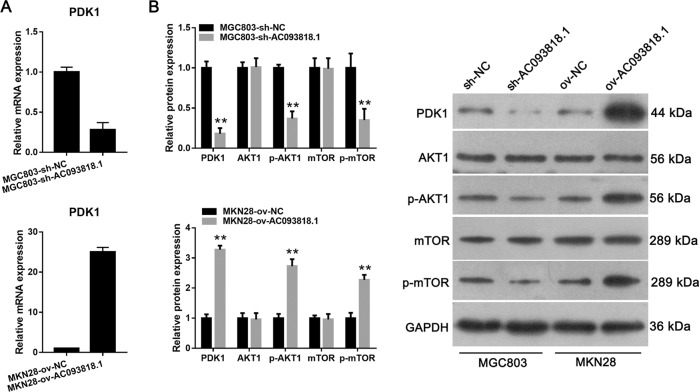
The effect of AC093818.1 on the expression of PDK1, AKT1, and mTOR. **a** The effect of AC093818.1 on the mRNA expression of PDK1. **b** The effect of AC093818.1 on the protein expression of PDK1, AKT1, p- AKT1, mTOR and p- mTOR. **P < 0.01.

The expression levels of PDK1, p-AKT1 and p-mTOR were obviously decreased in sh-AC093818.1-infected MGC803 cells relative to that in sh-NC-infected MGC803 cells. Moreover, PDK1, p-AKT1 and p-mTOR expression levels were significantly increased in ov-AC093818.1-infected MKN28 cells relative to that in ov-NC-infected MKN28 cells (Fig. 4). AC093818.1 silencing or overexpression had no obvious effect on total AKT1 and mTOR expression (Fig. 4). In addition, siRNA-4 transfection also downregulated expression levels of PDK1, p-AKT1 and p-mTOR (Supplementary Fig. 4).

### AC093818.1 binds to transcription factors STAT3 and SP1

**Fig. 5:**
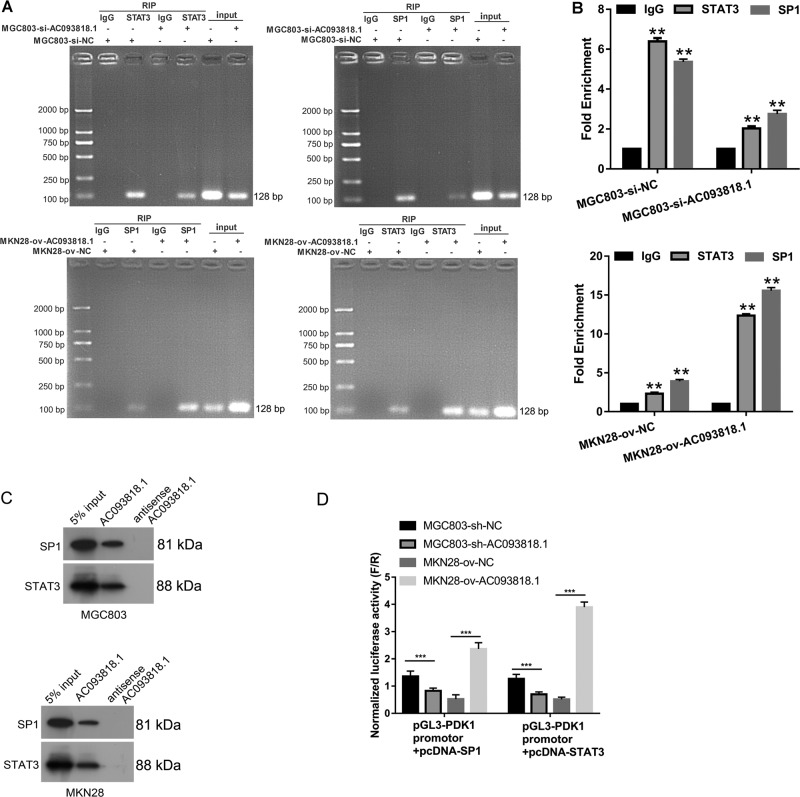
AC093818.1 can bind to transcription factors STAT3 and SP1. **a**, **b** RNA immunoprecipitation assay was carried out using sh-AC093818.1- and sh-NC-infected MGC803 cells, and ov-AC093818.1- and ov-NC-infected MKN28 cells. **a** AC093818.1 level was examined by qRT-PCR analysis and PCR products were resolved by 3% agarose gel electrophoresis. **b** The fold enrichment method (2^−ΔΔCt[ChIP/NIS])^ was used to calculated AC093818.1 level. **P < 0.01. **c** The protein levels of SP1 and STAT3 in RNA-binding proteins of labeled AC093818.1 enriched by RNA-protein pull-down assay using cell lysate of MGC803 and MKN28 cells. **d** AC093818.1 level can affect the transactivation effect of SP1 or STAT3 on PDK1 promotor. MGC803-sh-NC, MGC803-sh-AC093818.1, MKN28-ov-NC, MKN28-ov-AC093818.1 cells were transfected with luciferase reporter plasmid pGL3- PDK1 promotor along with control vector pRL-SV40, pcDNA-SP1, or pcDNA-STAT3. Cell lysates were prepared from cells after 48 h. Firefly luciferase activity was normalized with Renilla luciferase activity (F/R). ***P < 0.001.

The position of AC093818.1 transcript is chr2:172480840–172556529. The position of PDK1 gene is chr2:172,555,969–172,608,669. This position information indicated that AC093818.1 and PDK1 overlap on the promotor of PDK1. Because AC093818.1 can promote PDK1 mRNA expression (Fig. 4a), we predicted that AC093818.1 may be involved in transcriptional activation of PDK1 by recruiting transcription factors. It is reported that transcription factors STAT3 and SP1 have binding sites on the promotor of PDK1 and contribute to the transactivation of PDK1^23,24^. AC093818.1 could be detected in the RNA production purified by RIP assay using anti-SP1 or anti-STAT3 antibody as bait protein using cell lysate of sh-AC093818.1-infected MGC803 cells, sh-NC-infected MGC803 cells, ov-AC093818.1-infected MKN28 cells and ov-NC-infected MKN28 cells (Fig. 5a, b). In addition, SP1 and STAT3 protein could be detected in AC093818.1-binding proteins enriched by RNA-protein pull-down assay using cell lysate of MGC803 and MKN28 cells (Fig. 5c). These results revealed that AC093818.1 could bind to transcription factors STAT3 and SP1.

Moreover, we found that the normalized luciferase activity of pGL3-PDK1 promotor was decreased in sh-AC093818.1-infected MGC803 cells overexpressing SP1 or STAT3 compared to that in sh-NC-infected MGC803 cells overexpressing SP1 or STAT3 (Fig. 5d), and normalized luciferase activity of pGL3-PDK1 promotor was increased in ov-AC093818.1-infected MKN28 cells overexpressing SP1 or STAT3 compared to that in ov-NC-infected MKN28 cells overexpressing SP1 or STAT3 (Fig. 5d). The results of PDK1 promotor luciferase assay revealed that AC093818.1 level could affect the transactivated effect of SP1 or STAT3 on PDK1 promotor.

**Fig. 6:**
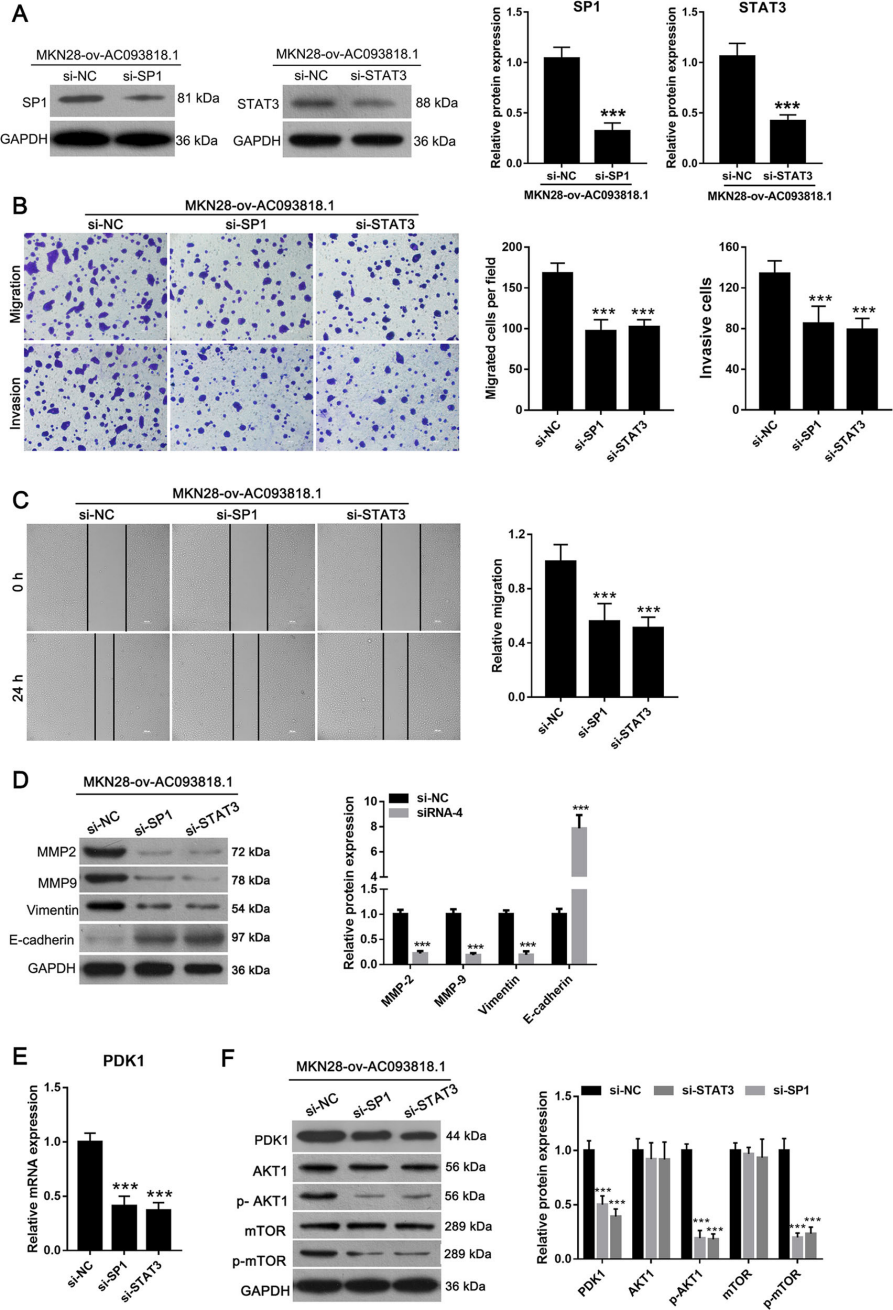
SP1 or STAT3 silencing inhibits the effect of AC093818.1 overexpression. **a** SP1 or STAT3 level in MKN28-ov-AC093818.1 cells after transfected with siRNA targeting SP1 (si-SP1) or STAT3 (si-STAT3), or negative control siRNA (si-NC). **b** Cell migration and invasion analyzed by the Transwell assay in MKN28-ov-AC093818.1 cells after transfected with si-SP1, si-STAT3, or si-NC. The amplification for the left representative images is ×200. **c** Cell migration analyzed by wound-healing assay in MKN28-ov-AC093818.1 cells after transfected with si-SP1, si-STAT3, or si-NC. The amplification for the left representative images is ×100. **d** Expression of MMP-2, MMP-9, vimentin, and E-cadherin analyzed by western blot in MKN28-ov-AC093818.1 cells after transfected with si-SP1, si-STAT3, or si-NC. **e** PDK1 mRNA level in MKN28-ov-AC093818.1 cells after transfected with si-SP1, si-STAT3, or si-NC. **f** Expression of PDK1, AKT1, p- AKT1, mTOR and p-mTOR analyzed by western blot in MKN28-ov-AC093818.1 cells after transfected with si-SP1, si-STAT3, or si-NC. ***P < 0.001.

SP1 or STAT3 silencing could alleviate the effect of AC093818.1 overexpression. si-SP1 or si-STAT3 was transfected into ov-AC093818.1-infected MKN28 cells to knockdown SP1 or STAT3 level (Fig. 6a). We found that si-SP1 or si-STAT3 transfection inhibited the migration and invasion of MGC803 cells (Fig. 6b, c), downregulated the expression levels of MMP-2, MMP-9, and vimentin, and upregulated the expression levels of E-cadherin compared to si-NC transfection in ov-AC093818.1-infected MKN28 cells (Fig. 6d). si-SP1 or si-STAT3 transfection also downregulated PDK1 mRNA level (Fig. 6e), and the protein levels of PDK1, p-AKT1 and p-mTOR (Fig. 6f).

## Discussion

GC is well-known for its metastatic and invasive properties. Current treatments for metastatic GC are very limited, and tremendous effort has been devoted to the development of novel targeted therapies. In the present study, AC093818.1 expression was demonstrated to be significantly higher in metastatic GC tissues and was significantly correlated with GC metastasis. In addition, AC093818.1 expression showed high sensitivity and specificity in the diagnosis of metastatic or nonmetastatic GC. Moreover, AC093818.1 promoted GC migration, invasion, and lung and liver metastases in vitro and in vivo.

The expression levels of natural antisense lncRNAs (AC093818.1, CTD-2541M15.1, BC047644, RP11-597M12.1, and RP11-40A13.1), especially AC093818.1, were significantly upregulated in metastatic GC samples relative to those in nonmetastatic GC samples. Our findings are consistent with those of Song et al.^17^, wherein microarray analysis indicated that the expression of these lncRNAs was significantly upregulated in lymph node-metastatic GC tissues relative to that in nonmetastatic GC tissues. Additionally, high expression of AC093818.1 was significantly correlated with invasion, lymphatic metastasis, distal metastasis, and TNM stage and showed high sensitivity and specificity for the diagnosis of metastatic or nonmetastatic GC. These findings indicate that high AC093818.1 expression is correlated with GC metastasis, highlighting the potential use of AC093818.1 as a predictive marker for GC diagnosis. However, the number of nonmetastatic GC samples in our study was much smaller than that of metastatic GC samples. The difference in the number of samples may affect the results. Thus, a larger number of nonmetastatic and metastatic GC samples are needed to confirm our results in future studies.

AC093818.1 is an antisense transcript of PDK1^17^, which is an oncogene known to promote tumorigenesis and metastasis^25,26^. Previous reports have indicated that antisense lncRNAs play important roles in tumorigenesis and metastasis^27–31^. However, the effects of natural antisense lncRNA AC093818.1 on tumorigenesis and metastasis remain unclear. In the present study, AC093818.1 overexpression promoted migration and invasion of GC cells and upregulated the expression of MMP-2 and MMP-9. On the other hand, AC093818.1 silencing inhibited GC cell migration and invasion, and downregulated the expression of MMP-2 and MMP-9 in vitro. Additionally, AC093818.1 overexpression promoted the metastasis of GC cells to the lungs and liver, and upregulated the expression of MMP-2 and MMP-9. By contrast, AC093818.1 silencing inhibited lung and liver metastasis of GC cells and downregulated the expression of MMP-2 and MMP-9 in vivo. Moreover, AC093818.1 overexpression upregulated E-cadherin expression and downregulated vimentin expression, whereas AC093818.1 silencing had the opposite effect both in vitro and in vivo indicating that AC093818.1 induced epithelial-to-mesenchymal transition (EMT) in GC. EMT plays a critical role in the invasive and metastatic potential of GC^32–34^. Therefore, we predict that AC093818.1 accelerates GC metastasis by inducing EMT. Our current findings implicate lncRNA AC093818.1 as a potential therapeutic target for GC metastasis.

The AKT/mTOR pathway is the key regulator of EMT^35^. AC093818.1 could increase the expression level of PDK1, which can activate the AKT/mTOR pathway by phosphorylating AKT1 at S473. The observations of the phosphorylation of AKT1 and mTOR support the view that AC093818.1 accelerates GC metastasis by increasing PDK1 level to activate the AKT/mTOR pathway. However, other signaling pathways may also participate in this regulation. In future studies, we will focus on the regulatory mechanisms studies by measuring the mRNA, ncRNA, and protein levels to further evaluate the underlying mechanism.

An increasing number of recent studies have shown that lncRNAs exhibit antisense binding to known protein-coding transcripts and act as natural antisense transcripts^36,37^. Natural antisense transcripts consist of the *cis*-subtype, which are transcribed from opposite DNA strands at the same genomic loci, and the *trans*-subtype, which are transcribed from distal loci^38^. Recent studies have verified that that natural antisense transcripts can be generated from pseudogenes^39^. Almost all antisense lncRNAs are localized in the nucleus and have been shown to regulate the expression of protein-coding genes in close genomic proximity (in *cis* or *cis*-acting) and target distant transcriptional complexes, such as activators or repressors (in *trans* or *trans*-acting), through various mechanisms^38^. The presently observed transcript overlap of PDK1 and AC093818.1 is at the promotor of PDK1. In addition, we found that AC093818.1 promoted PDK1 transcription. The transcription factors STAT3 and SP1 contributed to the transactivation of PDK1^23,24^. LncRNAs exert their roles via diverse mechanisms, such as cotranscriptional regulation. For example, lncTCF7 activates TCF7 expression by recruiting the SWI/SNF complex to the promoter of TCF7^40^. So, we predicted that AC093818.1 may be involved in transcriptional activation of PDK1 by recruiting the transcription factors STAT3 and SP1 to the PDK1 promotor. Our predication was verified by the results of RIP and RNA-protein pull-down assay, which showed that AC093818.1 can bind to transcription factors STAT3 and SP1. This is further supported by the observations that the level of AC093818.1 could affect the transactivation effect of SP1 or STAT3 on the PDK1 promotor, and that the SP1 or STAT3 levels also could influence the effect of AC093818.1 on the expression levels of PDK1, p-AKT1 and p-mTOR, and the capabilities of cell migration and invasion. However, besides transcription factors STAT3 and SP1, AC093818.1 may bind to other transcription factors to involve in regulating PDK1 transcription. More work is needed to fully illuminate the underlying mechanism.

The binding sites of SP1 or STAT3 on AC093818.1 were not identified. This is one limitation of our study. We plan to identify the binding sites of SP1 or STAT3 on AC093818.1 by deletion and mutation analysis of the AC093818.1 sequence.

In conclusion, lncRNA AC093818.1 promoted cell migration and invasion in vitro and in vivo and may thus be a potential predictive marker and therapeutic target for metastatic GC. The findings also provide novel insights into the mechanism underlying the role of AC093818.1 in promoting GC metastasis. Our collective results reveal that AC093818.1 increased the PDK1 level by transcriptional activation, which occurred by recruiting the transcription factors STAT3 and SP1 to the PDK1 promotor, resulting in the activation of the AKT/mTOR pathway to accelerate EMT-induced metastasis.

## Supplementary information

Supplementary Figure legends

Supplementary Table 1

Supplementary Figure 1

Supplementary Figure 2

Supplementary Figure 3

Supplementary Figure 4

## References

[1] Bray Freddie, Ferlay Jacques, Soerjomataram Isabelle, Siegel Rebecca L., Torre Lindsey A., Jemal Ahmedin (2018). Global cancer statistics 2018: GLOBOCAN estimates of incidence and mortality worldwide for 36 cancers in 185 countries. CA: A Cancer Journal for Clinicians.

[2] Torre LA (2015). Global cancer statistics, 2012. CA Cancer J. Clin..

[3] Chen W, Zheng R, Zeng H, Zhang S, He J (2015). Annual report on status of cancer in China, 2011. Chin. J. Cancer Res..

[4] Siegel RL, Miller KD, Jemal A (2018). Cancer statistics, 2018. CA Cancer J. Clin..

[5] Liu D (2016). The patterns and timing of recurrence after curative resection for gastric cancer in China. World J. Surg. Oncol..

[6] Yamashita K (2011). Trend in gastric cancer: 35 years of surgical experience in Japan. World J. Gastroenterol..

[7] Yang L, Froberg JE, Lee JT (2014). Long noncoding RNAs: fresh perspectives into the RNA world. Trends Biochem. Sci..

[8] Kogo R (2011). Long noncoding RNA HOTAIR regulates polycomb-dependent chromatin modification and is associated with poor prognosis in colorectal cancers. Cancer Res..

[9] Khorkova O, Hsiao J, Wahlestedt C (2015). Basic biology and therapeutic implications of lncRNA. Adv. Drug Deliv. Rev..

[10] Yin DD (2015). Decreased expression of long noncoding RNA MEG3 affects cell proliferation and predicts a poor prognosis in patients with colorectal cancer. Tumour Biol..

[11] Renganathan A, Felley-Bosco E (2017). Long noncoding RNAs in cancer and therapeutic potential. Adv. Exp. Med. Biol..

[12] Wang Y (2018). Long noncoding RNA Z38 promotes cell proliferation and metastasis and inhibits cell apoptosis in human gastric cancer. Oncol. Lett..

[13] Luo Y (2018). The long non-coding RNA LINC01606 contributes to the metastasis and invasion of human gastric cancer and is associated with Wnt/beta-catenin signaling. Int. J. Biochem. Cell Biol..

[14] Lian D, Amin B, Du D, Yan W (2017). Enhanced expression of the long non-coding RNA SNHG16 contributes to gastric cancer progression and metastasis. Cancer Biomark.: Sect. A Dis. Mark..

[15] Zhao J (2018). LncRNA PVT1 promotes angiogenesis via activating the STAT3/VEGFA axis in gastric cancer. Oncogene.

[16] Li Y (2017). Long non-coding RNA MALAT1 promotes gastric cancer tumorigenicity and metastasis by regulating vasculogenic mimicry and angiogenesis. Cancer Lett..

[17] Song W (2016). Identification of differentially expressed signatures of long non-coding RNAs associated with different metastatic potentials in gastric cancer. J. Gastroenterol..

[18] Guo H, Xia B (2016). Collapsin response mediator protein 4 isoforms (CRMP4a and CRMP4b) have opposite effects on cell proliferation, migration, and invasion in gastric cancer. BMC Cancer.

[19] Xu C (2017). SPP1, analyzed by bioinformatics methods, promotes the metastasis in colorectal cancer by activating EMT pathway. Biomed. Pharmacother..

[20] Pan Y, Jiao G, Wang C, Yang J, Yang W (2016). MicroRNA-421 inhibits breast cancer metastasis by targeting metastasis associated 1. Biomed. Pharmacother..

[21] Li, C. et al. Dysregulated lncRNA-UCA1 contributes to the progression of gastric cancer through regulation of the PI3K-Akt-mTOR signaling pathway. *Oncotarget*10.18632/oncotarget.19281 (2017). 10.18632/oncotarget.19281 PMC5706812 29212166

[22] Lopez-Bergami P (2010). c-Jun regulates phosphoinositide-dependent kinase 1 transcription: implication for Akt and protein kinase C activities and melanoma tumorigenesis. J. Biol. Chem..

[23] Xiao Q (2017). Activation of ERK and mutual regulation of Stat3 and SP1 contribute to inhibition of PDK1 expression by atractylenolide-1 in human lung cancer cells. Cell. Physiol. Biochem..

[24] Picco ME (2019). STAT3 enhances the constitutive activity of AGC kinases in melanoma by transactivating PDK1. Cell Biosci..

[25] Wang Z (2018). SOX9-PDK1 axis is essential for glioma stem cell self-renewal and temozolomide resistance. Oncotarget.

[26] Xia H (2018). EGFR-PI3K-PDK1 pathway regulates YAP signaling in hepatocellular carcinoma: the mechanism and its implications in targeted therapy. Cell Death Dis..

[27] Huang GW, Zhang YL, Liao LD, Li EM, Xu LY (2017). Natural antisense transcript TPM1-AS regulates the alternative splicing of tropomyosin I through an interaction with RNA-binding motif protein 4. Int. J. Biochem. Cell Biol..

[28] Peng B (2017). Silencing of lncRNA AFAP1-AS1 suppressed lung cancer development by regulatory mechanism in cis and trans. Oncotarget.

[29] Rinn JL (2007). Functional demarcation of active and silent chromatin domains in human HOX loci by noncoding RNAs. Cell.

[30] Wu Baihe, Chen Meizhu, Gao Minzhao, Cong Yunyan, Jiang Lifeng, Wei Jinqi, Huang Jin (2018). Down-regulation of lncTCF7 inhibits cell migration and invasion in colorectal cancer via inhibiting TCF7 expression. Human Cell.

[31] Zhang CL, Zhu KP, Ma XL (2017). Antisense lncRNA FOXC2-AS1 promotes doxorubicin resistance in osteosarcoma by increasing the expression of FOXC2. Cancer Lett..

[32] Wang JY (2018). Expression and clinical significance of autophagic protein LC3B and EMT markers in gastric cancer. Cancer Manag. Res..

[33] Zhou LH (2018). CircRNA_0023642 promotes migration and invasion of gastric cancer cells by regulating EMT. Eur. Rev. Med. Pharmacol. Sci..

[34] Cui H (2018). DNA methyltransferase 3A isoform b contributes to repressing E-cadherin through cooperation of DNA methylation and H3K27/H3K9 methylation in EMT-related metastasis of gastric cancer. Oncogene.

[35] Dong P (2014). The impact of microRNA-mediated PI3K/AKT signaling on epithelial-mesenchymal transition and cancer stemness in endometrial cancer. J. Transl. Med..

[36] Morris KV (2009). Long antisense non-coding RNAs function to direct epigenetic complexes that regulate transcription in human cells. Epigenetics.

[37] Latgé Guillaume, Poulet Christophe, Bours Vincent, Josse Claire, Jerusalem Guy (2018). Natural Antisense Transcripts: Molecular Mechanisms and Implications in Breast Cancers. International Journal of Molecular Sciences.

[38] Nie L (2012). Long non-coding RNAs: versatile master regulators of gene expression and crucial players in cancer. Am. J. Transl. Res..

[39] Muro EM, Andrade-Navarro MA (2010). Pseudogenes as an alternative source of natural antisense transcripts. BMC Evolut. Biol..

[40] Wang Y (2015). The long noncoding RNA lncTCF7 promotes self-renewal of human liver cancer stem cells through activation of Wnt signaling. Cell Stem Cell.

