# Supplementary material for: LncRNA AC093818.1 accelerates gastric cancer metastasis by epigenetically promoting PDK1 expression
Source: Cell Death Dis. 2020 Jan 27;11(1):64. doi: 10.1038/s41419-020-2245-2 (PMC6985138; doi:10.1038/s41419-020-2245-2)
Supplement: Supplementary file 1 — Supplementary Figure legends [file 41419_2020_2245_MOESM1_ESM.docx]

**Supplementary Figure Legends**

**Supplementary Fig. 1**. Expression levels of AC093818.1, CTD-2541M15.1, BC047644, RP11-597M12.1, and RP11-40A13.1 in 20 non-metastatic and metastatic GC samples measured by qRT-PCR. *P < 0.05, ** P < 0.01, and *** P < 0.001.

**Supplementary Fig. 2**. A: AC093818.1 expression in GC cells as determined by qRT-PCR. B: AC093818.1 expression in MGC803 cells transfected with the three AC093818.1-targeting siRNAs (siRNA-1, -2, -3) as determined by qRT-PCR. C: AC093818.1 expression in sh-AC093818.1-infected MGC803 cells and ov-AC093818.1-infected MKN28 cells as measured by qRT-PCR. D: AC093818.1 expression in MGC803 cells transfected with the three AC093818.1-targeting siRNAs (siRNA-4, -5, -6) as determined by qRT-PCR. ***P < 0.001.

**Supplementary Fig. 3**. The effect of AC093818.1-targeting siRNA-4 (siRNA-4) on migration and invasion of GC cells in vitro. A: Cell migration and invasion analyzed by the Transwell assay in MGC803 cells transfected with siRNA-4 or negative control siRNA (si-NC). The amplification for the left representative images is 200×. B: Cell migration analyzed by wound healing assay in MGC803 cells transfected with siRNA-4 or si-NC. The amplification for the left representative images is 100×. C: Expression of MMP-2, MMP-9, vimentin, and E-cadherin analyzed by western blot in MGC803 cells transfected with siRNA-4 or si-NC. ***P < 0.001.

**Supplementary Fig. 4**. The effect of AC093818.1-targeting siRNA-4 (siRNA-4) on the expression of PDK1, AKT1, and mTOR. A: The effect of siRNA-4 on the mRNA expression of PDK1. B: The effect of siRNA-4 on the protein expression of PDK1, AKT1, p- AKT1, mTOR and p- mTOR. ***P < 0.001.
