# Supplementary material for: LncRNA AC093818.1 accelerates gastric cancer metastasis by epigenetically promoting PDK1 expression
Source: Cell Death Dis. 2020 Jan 27;11(1):64. doi: 10.1038/s41419-020-2245-2 (PMC6985138; doi:10.1038/s41419-020-2245-2)
Supplement: Supplementary file 2 — Supplementary Table 1 [file 41419_2020_2245_MOESM2_ESM.docx]

**Supplementary Table 1.** Sequences of primers used in the current study.

| Gene | Primers |
| --- | --- |
| **Primer for PCR detection** |  |
| AC093818.1-F | 5ʹ GCTAGAGAAGCCACAGCCAGTA 3ʹ |
| AC093818.1-R | 5ʹ CCACACGTCCCAGTGTTCAG 3ʹ |
| BC047644-F | 5ʹ TTTGTGCTGGAAATGCTCTT 3ʹ |
| BC047644-R | 5ʹ ATAACCGTCTTGGCACTCTC 3ʹ |
| CTD-2541M15.1-F | 5ʹ GCCATGCGTAAAACTCCAAG 3ʹ |
| CTD-2541M15.1-R | 5ʹ TGCACACGTCACCAAGTTACT 3ʹ |
| RP11-40A13.1-F | 5ʹ GAATGTCCAGCATGATCTCCA 3ʹ |
| RP11-40A13.1 -R | 5ʹ CAGCTAGGAAACCAGAGTGAT 3ʹ |
| RP11-597M12.1-F | 5ʹ GCACCACCAGCATCACCACAC 3ʹ |
| RP11-597M12.1-R | 5ʹ GCACCGCCCTCTGACATGAC 3ʹ |
| β-actin-F | 5ʹ TGGATCAGCAAGCAGGAGTA 3ʹ |
| β-actin-R | 5ʹ TCGGCCACATTGTGAACTTT 3ʹ |
| **Primers for PCR amplification** |  |
| AC093818.1XhoIF | 5ʹccgctcgagTCCCGCGGCCCAGCTGGTTTGTTTTC 3ʹ |
| AC093818.1BamHIF | 5ʹcgcggatccTCCTATGCAGCACATGATTTTTATTG 3ʹ |
