# Supplementary figures and images for: LncRNA AC093818.1 accelerates gastric cancer metastasis by epigenetically promoting PDK1 expression
Source: Cell Death Dis. 2020 Jan 27;11(1):64. doi: 10.1038/s41419-020-2245-2 (PMC6985138; doi:10.1038/s41419-020-2245-2)

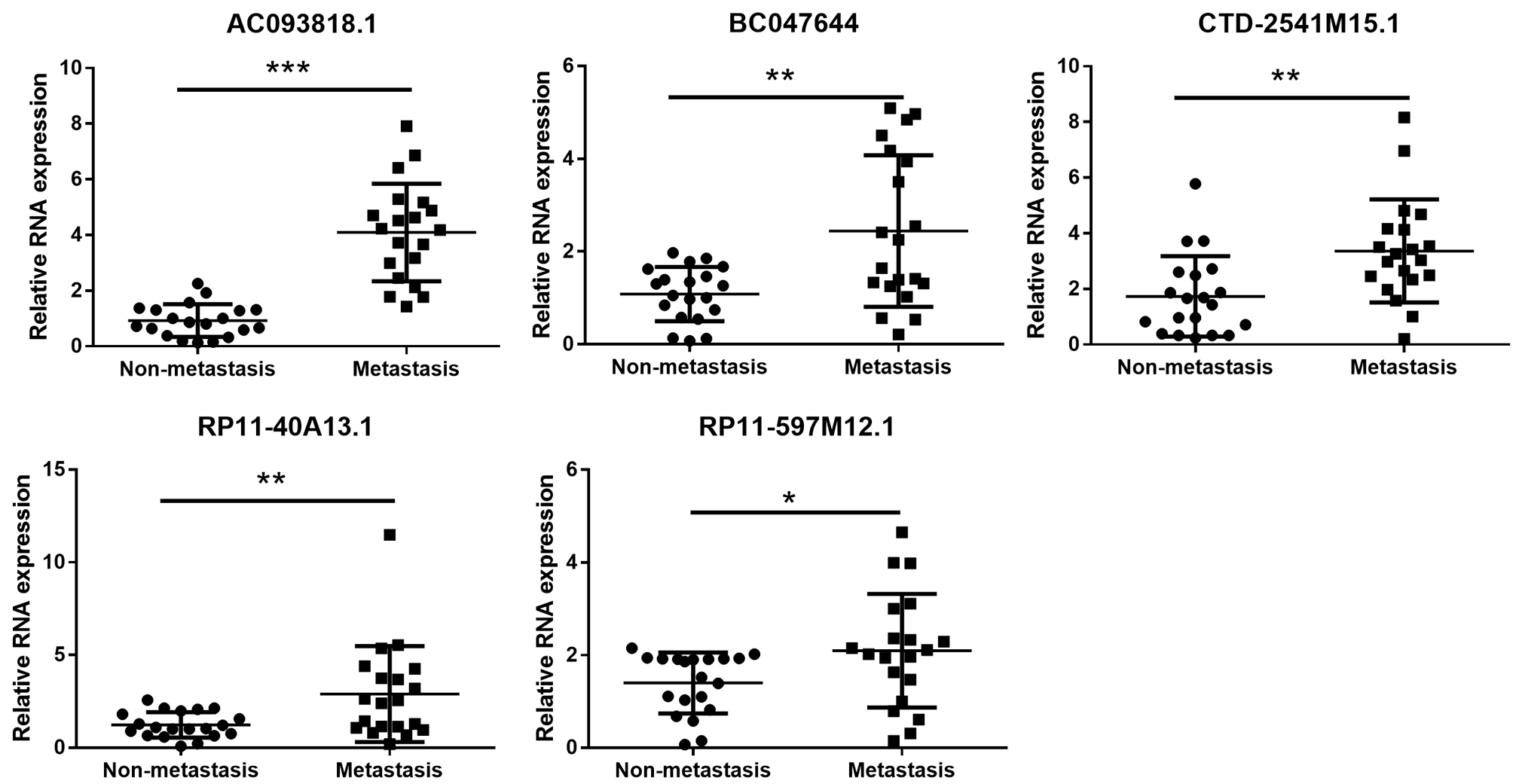

Supplement: Supplementary file 3 — Supplementary Figure 1 [file 41419_2020_2245_MOESM3_ESM.tif]

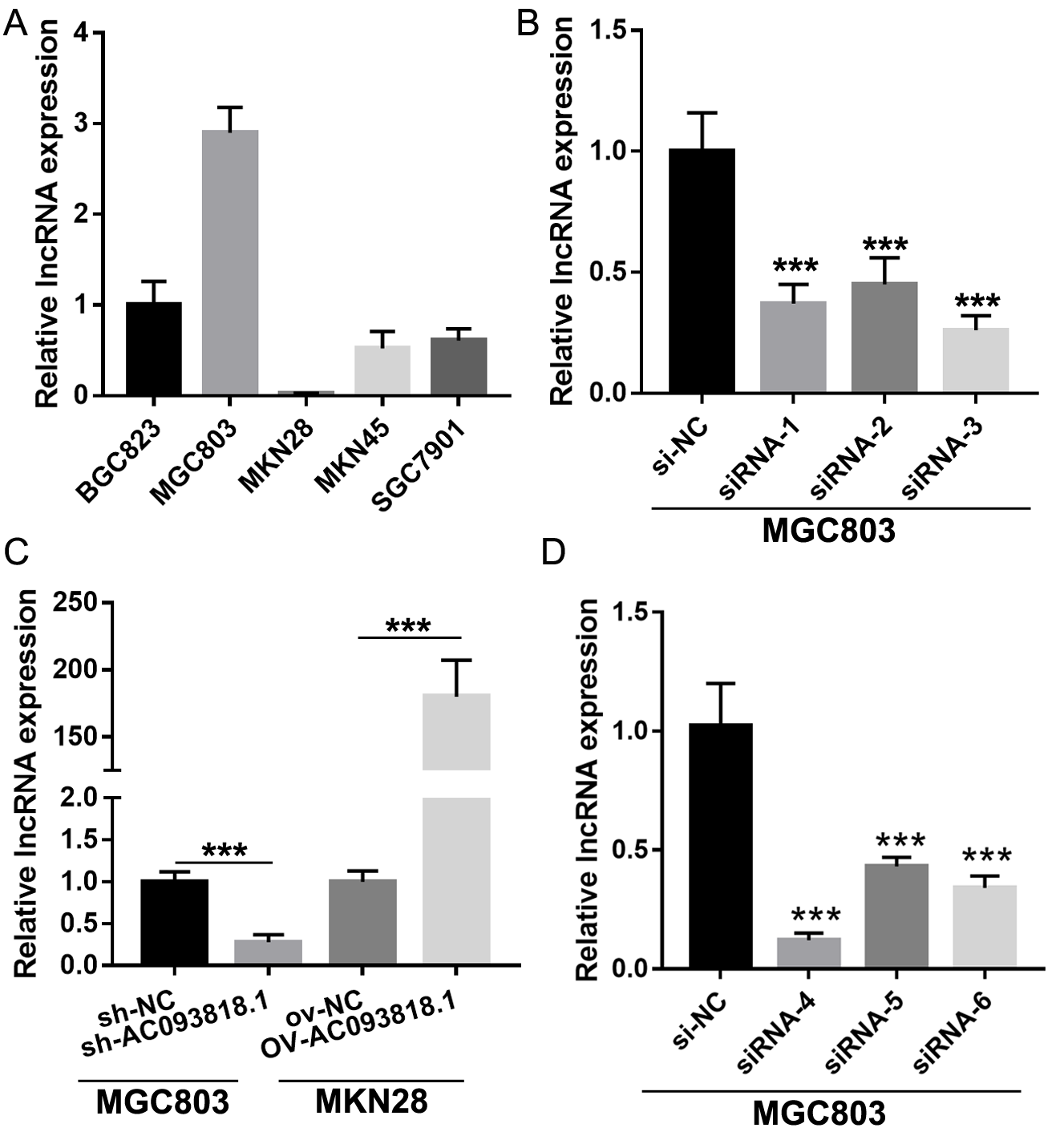

Supplement: Supplementary file 4 — Supplementary Figure 2 [file 41419_2020_2245_MOESM4_ESM.tif]

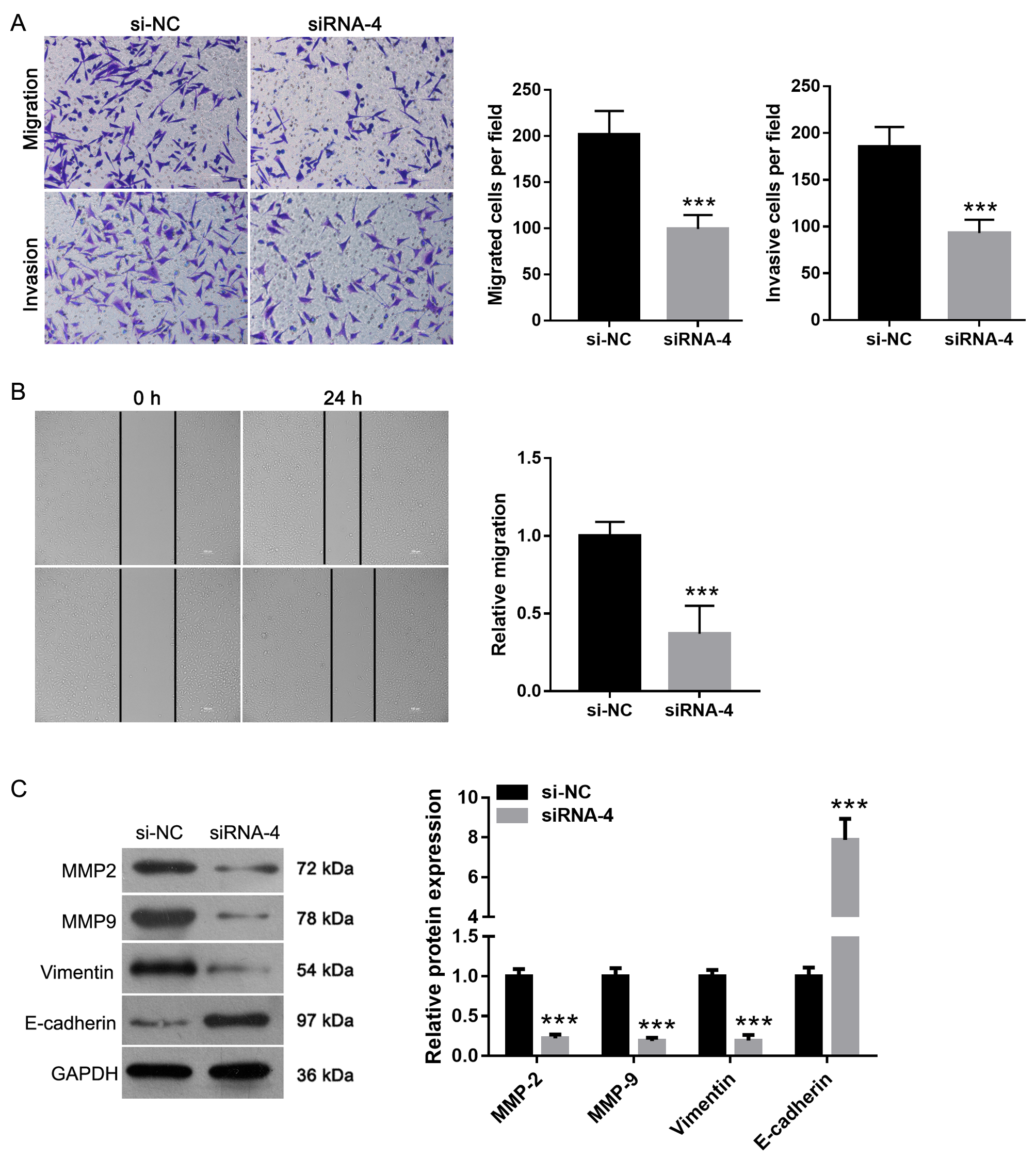

Supplement: Supplementary file 5 — Supplementary Figure 3 [file 41419_2020_2245_MOESM5_ESM.tif]

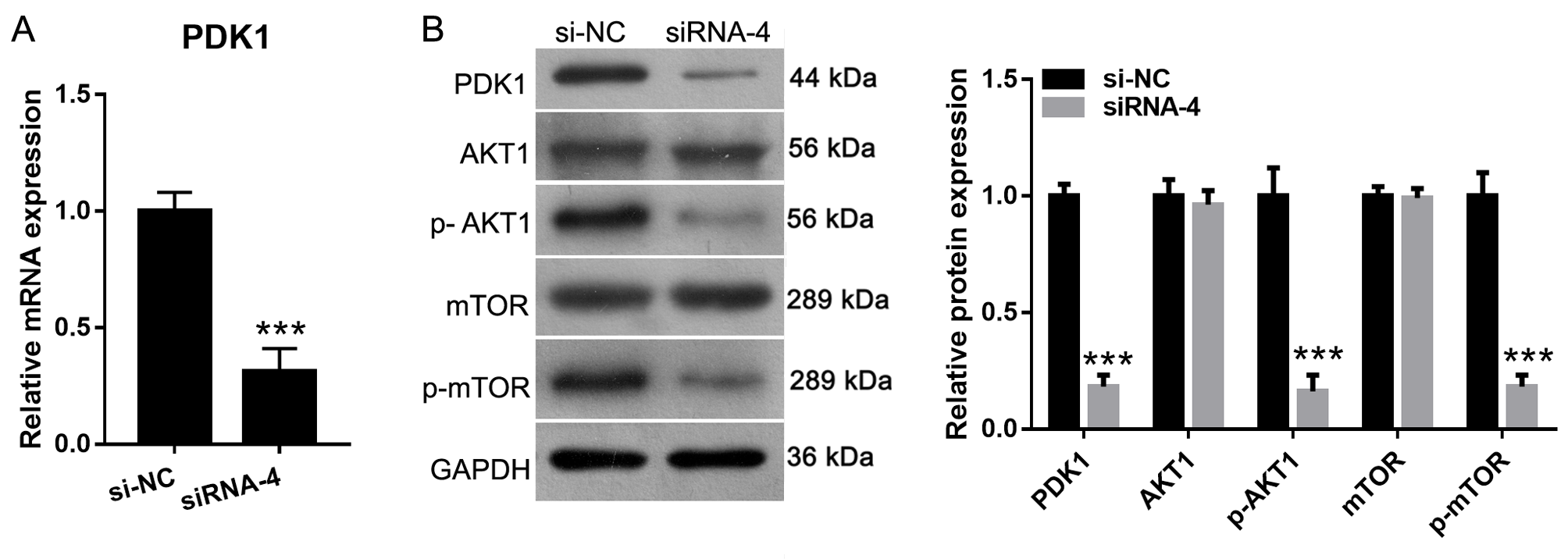

Supplement: Supplementary file 6 — Supplementary Figure 4 [file 41419_2020_2245_MOESM6_ESM.tif]
